# Association of deranged cerebrovascular reactivity with brain injury following cardiac arrest: a post-hoc analysis of the COMACARE trial

**DOI:** 10.1186/s13054-021-03764-6

**Published:** 2021-09-28

**Authors:** Johanna Laurikkala, Anders Aneman, Alexander Peng, Matti Reinikainen, Paul Pham, Pekka Jakkula, Johanna Hästbacka, Erika Wilkman, Pekka Loisa, Jussi Toppila, Thomas Birkelund, Kaj Blennow, Henrik Zetterberg, Markus B. Skrifvars

**Affiliations:** 1 Department of Anaesthesiology, Intensive Care and Pain Medicine, University of Helsinki and Helsinki University Hospital, Meilahden SairaalaHaartmaninkatu 4, 000290 Helsinki, Finland; 2 Intensive Care Unit, Liverpool Hospital, South Western Sydney Local Health District, Sydney, Australia; 3 Faculty of Medicine, The University of New South Wales, Sydney, Australia; 4 Faculty of Medicine and Health Sciences, Macquarie University, Sydney, Australia; 5 Department of Anaesthesiology and Intensive Care, University of Eastern Finland and Kuopio University Hospital, Kuopio, Finland; 6 Dept of Anaesthesia, John Hunter Hospital, Newcastle, NSW Australia; 7 Department of Intensive Care, Päijät-Häme Central Hospital, Lahti, Finland; 8 Department of Neurology, University of Helsinki and Helsinki University Hospital, Helsinki, Finland; 9 Aarhus University Hospital, Aarhus, Denmark; 10 Department of Psychiatry and Neurochemistry, Institute of Neuroscience and Physiology, The Sahlgrenska Academy at the University of Gothenburg, Mölndal, Sweden; 11 Clinical Neurochemistry Laboratory, Sahlgrenska University Hospital, Mölndal, Sweden; 12 DUK Dementia Research Institute at UCL, London, UK; 13 Department of Neurodegenerative Disease, UCL Institute of Neurology, London, UK; 14 Department of Emergency Care and Services, Helsinki University Hospital and University of Helsinki, Helsinki, Finland

**Keywords:** Cerebrovascular reactivity, Out-of-hospital cardiac arrest, Hypoxic-ischaemic brain injury

## Abstract

**Background:**

Impaired cerebrovascular reactivity (CVR) is one feature of post cardiac arrest encephalopathy. We studied the incidence and features of CVR by near infrared spectroscopy (NIRS) and associations with outcome and biomarkers of brain injury.

**Methods:**

A post-hoc analysis of 120 comatose OHCA patients continuously monitored with NIRS and randomised to low- or high-normal oxygen, carbon dioxide and mean arterial blood pressure (MAP) targets for 48 h. The tissue oximetry index (TO_x_) generated by the moving correlation coefficient between cerebral tissue oxygenation measured by NIRS and MAP was used as a dynamic index of CVR with TO_x_ > 0 indicating impaired reactivity and TO_x_ > 0.3 used to delineate the lower and upper MAP bounds for disrupted CVR. TO_x_ was analysed in the 0–12, 12–24, 24–48 h time-periods and integrated over 0–48 h. The primary outcome was the association between TO_x_ and six-month functional outcome dichotomised by the cerebral performance category (CPC1-2 good vs. 3–5 poor). Secondary outcomes included associations with MAP bounds for CVR and biomarkers of brain injury.

**Results:**

In 108 patients with sufficient data to calculate TO_x_, 76 patients (70%) had impaired CVR and among these, chronic hypertension was more common (58% vs. 31%, *p* = 0.002). Integrated TO_x_ for 0–48 h was higher in patients with poor outcome than in patients with good outcome (0.89 95% CI [− 1.17 to 2.94] vs. − 2.71 95% CI [− 4.16 to − 1.26], *p* = 0.05). Patients with poor outcomes had a decreased upper MAP bound of CVR over time (*p* = 0.001), including the high-normal oxygen (*p* = 0.002), carbon dioxide (*p* = 0.012) and MAP (*p* = 0.001) groups. The MAP range of maintained CVR was narrower in all time intervals and intervention groups (*p* < 0.05). NfL concentrations were higher in patients with impaired CVR compared to those with intact CVR (43 IQR [15–650] vs 20 IQR [13–199] pg/ml, *p* = 0.042).

**Conclusion:**

Impaired CVR over 48 h was more common in patients with chronic hypertension and associated with poor outcome. Decreased upper MAP bound and a narrower MAP range for maintained CVR were associated with poor outcome and more severe brain injury assessed with NfL.

*Trial registration* ClinicalTrials.gov, NCT02698917.

**Supplementary Information:**

The online version contains supplementary material available at 10.1186/s13054-021-03764-6.

## Background

Out-of-hospital cardiac arrest (OHCA) carries a high overall mortality rate related to hypoxic-ischaemic brain injury (HIBI) [1]. On suggested mechanism of HIBI is ongoing cerebral hypoxia related to insufficient cerebral blood flow (CBF) after return of spontaneous circulation (ROSC) [2–6]. Cerebrovascular reactivity (CVR) refers to the ability of the brain vasculature to change flow resistance in response to fluctuating blood tension of oxygen and carbon dioxide and varying mean arterial blood pressure (MAP) levels, aiming to maintain a relatively constant CBF [7, 8]. The specific aspect of CVR related to MAP is commonly referred to as cerebral autoregulation. Near-infrared spectroscopy (NIRS) may be used to monitor spontaneous low-frequency oscillations in cerebral tissue oxygenation (cStO_2_) that reflect CBF and, when correlated with simultaneous changes in MAP, allow for the tissue oxygenation index (TO_x_) to be derived as an index of dynamic CVR. This technique has known limitations but provides a non-invasive method to estimate CVR at the bedside [9–12]. Impaired CVR monitored by TO_x_ is associated with poor neurological outcomes in acute neurocritical conditions [13, 14] and in OHCA, as suggested by smaller, single-centre, observational cohort studies [4, 5, 15].

In the multicentre randomised controlled pilot study COMACARE, 120 adult comatose mechanically ventilated survivors of OHCA with an initial rhythm of ventricular fibrillation/tachycardia were treated with low- or high-normal targets for MAP, arterial oxygen (P_a_O_2_) and carbon dioxide (P_a_CO_2_) tension [16]. All patients were monitored with the same type of NIRS device over the first 48 h of ICU care enabling an estimation of CVR over time. The primary outcome in this post-hoc analysis was the association between TOx and six-month neurological outcome dichotomised by cerebral performance category (CPC1-2 good vs. 3–5 poor), and we hypothesised that CVR would be impaired in patients with poor outcome.

## Methods

### Setting and participants

Six ICUs in Finland and one in Denmark participated in the COMACARE study that was conducted from March 2016 to March 2017. The study protocol and main results have been published earlier [16–18]. Briefly, the study included 120 comatose, mechanically ventilated survivors resuscitated from witnessed OHCA with ventricular tachycardia or fibrillation as the initial rhythm. The trial used a 2^3^ factorial design where each patient was randomised into one of eight arms, each having a different combination of targets: a low-normal (65–75 mmHg) or high-normal (80–100 mmHg) MAP, a normal (10–15 kPa) or moderately elevated (20–25 kPa) P_a_O_2_ and a low-normal (4.5–4.7 kPa) or high-normal (5.8–6.0 kPa) P_a_CO_2_. All patients were treated with targeted temperature management at either 33 °C or 36 °C, and all patients had invasive blood pressure monitoring. The protocol for this post-hoc analysis was published prior to commencing the study [19].

### Patient data and monitoring of cerebrovascular reactivity

Patient demographics, cardiac arrest and resuscitation characteristics, ICU acuity (APACHE II) and treatment factors were captured. The cStO_2_ was measured using the INVOS 5100C monitor (Covidien Company, USA) with two skin sensors attached bilaterally to the patient’s forehead, avoiding the frontal sinuses and temporal muscles. The mean values of the left and right hemispheric cStO_2_ values were used to calculate the TO_x_ as a moving Pearson correlation coefficient in the time domain between 10 min averages of MAP and cStO_2_ using the ICM + Brain Monitoring software (Version 8.3, University of Cambridge) [9]. A TO_x_ within the − 1 to 0 range was used to indicate maintained CVR, and a TO_x_ from > 0 to 1 indicated impaired CVR. Mean TO_x_ values were derived for three time periods: 0–12 h, 12–24 h and 24–48 h. The TO_x_ values were subsequently divided into MAP bins of 5 mmHg and fitted to a second-order polynomial with its nadir determining the optimal observed TO_x_ (OptTO_x_) [20]. The OptMAP was determined as the MAP corresponding to the OptTO_x_ on the fitted curve. The MAP intersecting a TO_x_ threshold of 0.30 on the second-order polynomial fit was calculated for each patient to represent the lower and upper bounds of MAP for maintained CVR and the range of MAP with maintained CVR calculated as the difference [19]. The nomenclature cerebrovascular reactivity rather than cerebral autoregulation is used throughout this paper to reflect the concomitant MAP, PaO2 and PaCO2 targets studied.

### Biomarkers of brain injury

Serum samples were collected at 48 h after OHCA and concentrations of neuron specific enolase (NSE), the astrocytic calcium-binding protein S100B and neurofilament light (NfL) were measured as previously described [21].

### Neurological outcome

An independent neurologist blinded to the study interventions evaluated the patients’ functional outcomes six months after OHCA. Neurological outcomes were assessed according to Pittsburgh Cerebral Performance Categories (CPC) [22]. Good outcome was defined as CPC 1–2 (good cerebral performance or moderate cerebral disability) and poor outcome as CPC 3–5 (severe cerebral disability, coma/vegetative state or death).

### Study outcomes

The primary outcome was the association between impaired CVR as determined by TOx and neurological outcome. The secondary outcomes were associations between CVR characteristics (OptTOx, OptMAP, the upper and lower MAP bounds as well as the MAP range for maintained CVR) and neurological status and biomarkers of brain injury. The primary and secondary outcomes were stratified by the MAP, PaO2 and PaCO2 targets according to the COMACARE study design. We also explored factors associated with impaired CVR including patient, resuscitation and ICU admission characteristics.

### Statistical methods

Categorical data are presented as counts and percentages and compared with the chi-square test. Continuous data are presented as medians with interquartile ranges (IQR) and compared with the Mann–Whitney U-test. We performed a univariable analysis to determine possible risk factors for impaired CVR defined as TO_x_ > 0 and poor neurological outcome. We calculated the time-integrated area below OptTO_x_ and OptMAP and outside the upper and lower MAP bounds for maintained CVR as well as the MAP range (mmHg min). Both the mean TOx in the separate time periods and integrated over 0–48 h were evaluated. Comparisons of TO_x_, OptTO_x_, OptMAP, MAP bounds and range for maintained CVR in the time periods 0–12 h, 12–24 h and 24–84 h between outcome groups were performed using mixed linear model analyses. We constructed multivariable models including age, sex, time to ROSC and hypertension as factors likely to influence CVR. All statistical analyses were performed using SPSS 25 (IBM, Armonk, NY, USA) or GraphPad PRISM (version 7.0d for MacOSX, GraphPad Software, La Jolla, CA, USA). A *p* value < 0.05 was considered statistically significant.

## Results

**Fig. 1:**
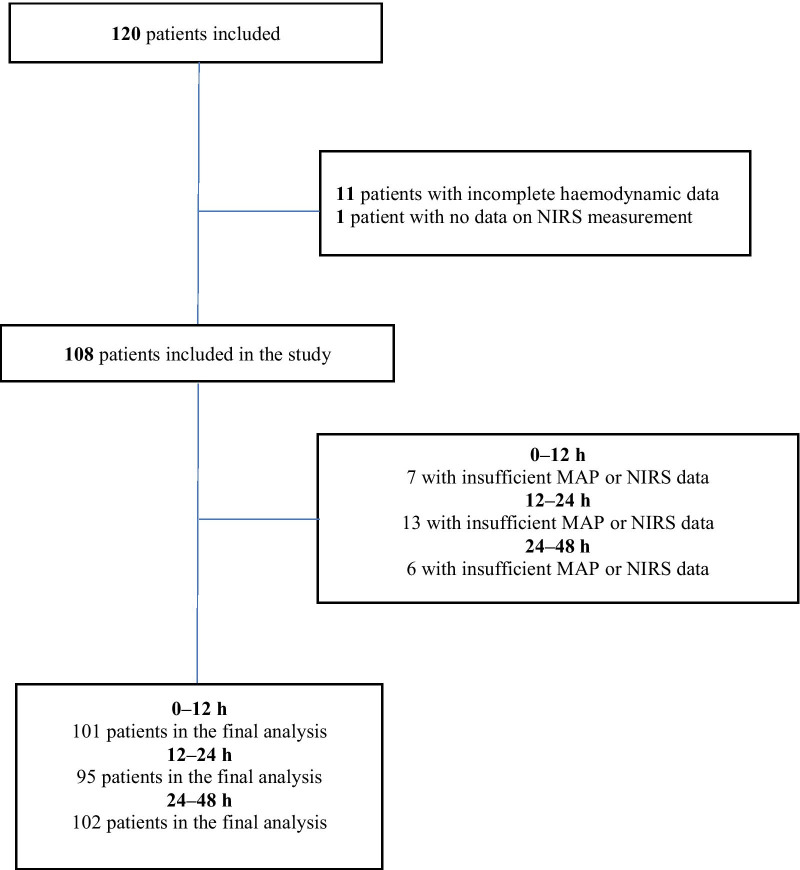
Flowchart of excluded and included study patients

From the main COMACARE study, we excluded 12 survivors with incomplete registrations of MAP or cStO_2_ data. A flowchart of study patients is presented in Fig. 1. Of the 108 OHCA patients, 32 had intact and 76 had impaired CVR. Baseline characteristics, cardiac arrest and resuscitation factors, medical history and ICU care indexed by whether the CVR was maintained or not are presented in Table 1. Patients with chronic hypertension were more likely to have impaired CVR (57.9% vs 31.3%, *p* = 0.012). In a multivariable model including age, gender and time to ROSC, chronic hypertension (OR 2.95, 95% CI [1.16, 7.51], *p* = 0.024) was the only independent predictor of impaired CVR.

**Table 1:** Patient characteristics with maintained (TOx < 0) or impaired (TOx > 0) cerebrovascular reactivity (CVR)

Characteristic	Data available	All n = 108	Maintained CVR n = 32	Impaired CVR n = 76	*p*-value
Gender, n% (male)	108	90 (83.3%)	24 (75.0%)	66 (73.3%)	0.133
Age, years	108	61.0 (53.0–70.0)	55.0 (44.5–69.0)	61.0 (53.0–66.3)	0.057
BMI	106	26.3 (23.4–29.4)	26.3 (24.2–28.6)	26.1 (23.3–29.4)	0.866
Neurologic function before cardiac arrest	108				0.950
Normal, CPC1, n (%)		101 (93.5%)	30 (93.8%)	71 (93.4%)	
Some disability, CPC2, n (%)		7 (6.5%)	2 (6.3%)	5 (6.6%)	
Cardiac arrest characteristics and resuscitation factors					
Bystander CPR or compressions only, n (%)	108	88 (81.5%)	25 (78.1%)	63 (82.9%)	0.564
Initial rhythm	108				0.359
VF, n (%)		106 (98.1%)	32 (100.0%)	74 (97.4%)	
VT, n (%)		2 (1.9%)	0 (0%)	2 (2.6%)	
Time to BLS, min	106	7.0 (6.0–9.0)	7.0 (6.0–10.0)	8.0 (6.0–9.3)	0.813
Time to ALS, min	106	10.0 (8.0–12.0)	9.0 (7.5–12.0)	10.0 (8.0–12.0)	0.643
Time to ROSC, min	106	21.0 (16.0–25.0)	17.7 (15.5–25.0)	18.8 (14.1–24.5)	0.197
GCS after ROSC	99	3.0 (3.0–3.0)	3.0 (3.0–4.5)	3.0 (3.0–3.3)	0.093
Medical history					
IHD (NYHA class IV), n (%)	107	2 (1.9%)	0 (0%)	2 (2.7%)	0.353
HTA, n (%)	108	54 (50.0%)	10 (31.3%)	44 (57.9%)	0.012
Smoker, n (%)	95	32 (33.7%)	10 (37.0%)	22 (32.4%)	0.665
Treatment					
PCI before ICU admission	108	53 (49.1%)	14 (43.8%)	39 (51.3%)	0.477
Severity of illness score					
Apache II	108	27.0 (24.0–30.0)	27.0 (24.0–29.0)	27.0 (24.0–31.3)	0.119
Targeted temperature management	108				0.140
33 °C, n (%)		77 (71.3%)	26 (34.0%)	51 (66.0%)	
36 °C, n (%)		31 (28.7%)	6 (20.0%)	25 (80.0%)	
EEG – beginning POOR	107	81 (75.7%)	24 (77.4%)	57 (75.0%)	0.794
EEG – end POOR	107	36 (33.6%)	9 (29.0%)	27 (35.5%)	0.523
Brain oedema, NO	108	108 (100%)	32 (100%)	76 (100%)	
Duration of mechanical ventilation, hours	80	70.5 (49.5–95.2)	70.5 (49.1–90.5)	63.1 (49.2–95.1)	0.637
Length of stay in ICU, hours	104	103.0 (76.6–145.9)	107.0 (78.5–132.0)	92.30 (75.0–141.0)	0.575
Mortality 30 d	108	33 (30.6%)	8 (25.0%)	25 (32.9%)	0.418
CPC 6 months, poor	108	35 (32.4%)	9 (28.1%)	26 (34.2%)	0.539

Numbers are median (interquartile range) or n (percentage)

*CVR* cerebrovascular reactivity, *BMI* body mass index (kg/m^2^), *BLS* basic life support, *ALS* advanced life support, *GCS* Glasgow coma scale, *ROSC* return of spontaneous circulation, *IHD* ischemic heart disease, *HTA* arterial hypertension, *PCI* percutaneous coronary intervention

### Markers of cerebrovascular reactivity

The primary outcome i.e. the comparison of changes in CVR assessed by TOx against neurological outcome are shown in Table 2. The primary outcome variable mean TOx was higher in patients with poor functional outcome when analysed with mixed linear analysis (*p* = 0.004). In addition, the cumulative time-integrated “AUROC” TOx (0–48 h) was higher in patients with poor neurological outcome than in those with good outcome (0.89 (95% CI [− 1.17; 2.94] vs − 2.71 (95% CI [− 4.16; − 1.26], *p* = 0.05).

**Table 2:** Mixed linear model analysis for Mean TOx, OptTOx, OptMAP, upper and lower MAP bounds and MAP range for maintained CVR in three time periods: 0–12 h, 12–24 h and 24–48 h

	Group	0–12 h	12–24 h	24–48 h	*p*-value
Mean TOx	Poor outcome	0.06 (− 0.07 to 0.14)	− 0.03 (− 0.11 to 0.16)	0.01 (− 0.09 to 0.14)	0.004
	Good outcome	− 0.04 (− 0.12 to 0.04)	− 0.05 (− 0.21 to 0.05)	− 0.02 (− 0.10 to 0.06)	
OptTOx	Poor outcome	− 0.21 (− 0.35 to − 0.02)	− 0.17 (− 0.36 to − 0.02)	− 0.19 (− 0.36 to − 0.05)	0.077
	Good outcome	− 0.24 (− 0.38 to − 0.14)	− 0.27 (− 0.48 to − 0.10)	− 0.23 (− 0.37 to − 0.11)	
OptMAP	Poor outcome	79.7 (73.5–87.8)	76.4 (71.1–81.7)	81.7 (72.4–88.3)	0.079
	Good outcome	80.9 (71.4–86.7)	81.7 (73.0–87.4)	82.7 (75.0–87.4)	
Upper MAP bound	Poor outcome	90.5 (81.1–98.4)	89.5 (80.6–92.9)	92.8 (83.4–100.3)	0.001
	Good outcome	94.7 (85.4–102.3)	93.4 (83.2–100.4)	94.4 (89.0–101.0)	
Lower MAP bound	Poor outcome	69.6 (64.2–74.4)	66.2 (63.0–73.1)	68.5 (63.4–74.4)	0.569
	Good outcome	68.0 (63.3–75.9)	69.7 (62.1–74.7)	68.4 (63.6–73.5)	
MAP range	Poor outcome	19 (15–30)	24 (17–26)	23 (17–29)	< 0.001
	Good outcome	28 (23–36)	25 (20–36)	33 (25–39)	

*p* values are comparing good versus poor neurologic outcome

Values are expressed as medians (interquartile ranges)

*OptTOx* optimal tissue oxygenation index

*OptMAP* optimal mean arterial pressure

With regard to the secondary outcomes, the mean TOx, OptTO_x_, OptMAP, upper and lower MAP bounds and MAP range for maintained CVR in patients for time periods 0–12 h, 12–24 h and 24–48 h are reported in Table 2. Patients with poor neurological function had a decreased upper MAP bound for maintained CVR compared to patients with good neurological function in all time intervals in the mixed linear model analysis (*p* = 0.001) (Table 2). In the intervention groups, the upper MAP bound for maintained CVR was significantly decreased during all three time periods in patients with poor neurological outcomes compared to patients with good outcomes in the high-normal P_a_CO_2_ (*p* = 0.012), high P_a_O_2_ (*p* = 0.002) and high-normal MAP (*p* = 0–001) groups (Additional file 1: Table S1). The MAP range of maintained CVR was narrower in patients with poor outcomes in all three time periods in the mixed linear analysis (*p* < 0.001) (Table 2). We found no difference in OptTO_x_, lower MAP bound and OptMAP between patients with good or poor outcomes (Table 2).

### Time-integrated mean arterial pressure area between, below and above the bounds for maintained cerebrovascular reactivity

**Fig. 2:**
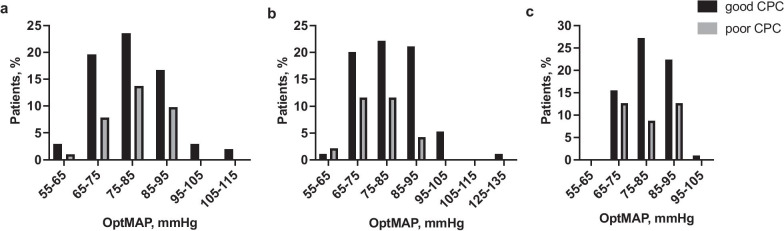
Optimal MAP (OptMAP) in three time periods: **a** 0–12 h, **b** 12–24 h and **c** 24–48 h, with good or poor six-month neurologic outcomes

There were no statistically significant differences in the MAP areas between the lower and upper MAP bounds for maintained CVR nor below the lower MAP bound or above the upper MAP bound comparing patients with good or poor long-term outcomes (Table 3). Also, MAP areas were explored within all treatment arms (Additional file 1: Table S2). The MAP area below the lower MAP bound for maintained CVR was larger in the low P_a_CO_2_ group during the time periods 12–24 h and 24–48 h (*p* = 0.024) (Additional file 1: Table S2). Patients in the high MAP group had a larger MAP area below the lower MAP bound for maintained CVR during the time periods 0–12 h and 12–24 h (*p* = 0.021) (Additional file 1: Table S2). A comparison of optimal MAP groups in all time periods, 0–12 h, 12–24 h and 24–48 h, is shown in Fig. 2.

**Table 3:** Mixed linear analysis for time-integrated mean arterial pressure (MAP) area (mmHg min) between the lower and upper MAP bounds for maintained cerebrovascular reactivity (CVR) and total MAP area below the lower and above the upper MAP bound for maintained CVR stratified by the factorial design groups during three time periods: 0–12 h, 12–24 h and 24–48 h, with good or poor neurologic outcomes

	0–12 h	12–24 h	24–48 h	*p*-value
MAP area between lower and upper MAP bounds, mmHg min				
All patients	5399 (3950–8231)	7958 (4818–11079)	15870 (9423–21785)	
Good outcome	6900 (4849–8880)	8389 (5166–11479)	17277 (10180–22792)	0.246
Poor outcome	5542 (3292–6903)	6134 (3500–9262)	12139 (6305–16794)	
MAP area below lower MAP bound, mmHg min				
All patients	132 (36–479)	107 (31–305)	235 (6–653)	
Good outcome	118 (33–358)	103 (28–319)	235 (54–503)	0.061
Poor outcome	150 (46–502)	134.6 (54.2–358.3)	197 (85–1153)	
MAP area above upper MAP bound, mmHg min				
All patients	315 (100–846)	195 (55–1004)	266 (99–101)	
Good outcome	319 (96–800)	135 (28–338)	222 (79–1011)	0.243
Poor outcome	314 (108–1369)	253 (64–10492)	339 (149–1010)	

Values are expressed as medians (interquartile ranges)

### NSE, S100B and NfL and cerebrovascular reactivity

**Fig. 3:**
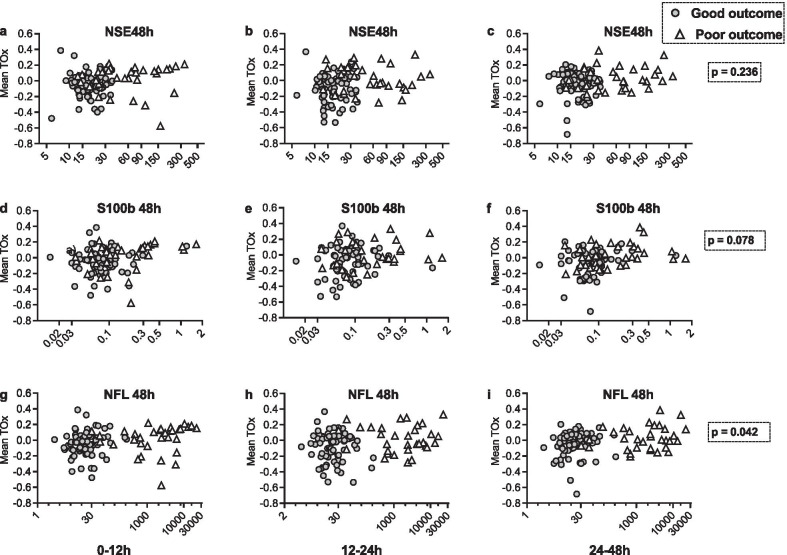
**a**–**c** 48 h neuron-specific enolase (NSE) concentrations ($\mathrm{\mu g}/\mathrm{l}$), **d**–**f** 48 h S100B concentrations ($\mathrm{\mu g}/\mathrm{l}$) and **g**–**i** 48 h neurofilament (NfL) concentrations (pg/ml) against mean cerebrovascular reactivity (mean TOx) during the time intervals 0–12 h, 12–24 h and 24–48 h. Patients are classified according to good or poor neurologic outcomes. Data is presented with logharitmic X-axes

Biomarker concentrations 48 h after OHCA versus CVR are illustrated in Fig. 3. The NfL concentration was higher in patients with impaired CVR compared with those with maintained CVR (43.0 [inter-quartile range, 15.2–650.3] µg/L vs. 20.4 [13.0–199.3] µg/L). A mixed linear model of NfL levels over time in patients with maintained or impaired CVR showed a statistically significant difference (*p* = 0.042). No significant differences in the median serum NSE or S100B concentrations were found after 48 h in OHCA patients based on whether they had maintained or impaired CVR (Fig. 3).

The analysis of all biomarkers at 48 h indexed by whether CVR was maintained or impaired in the different study time periods indexed by intervention groups is reported in Additional file 1: Table S3 and S4. Biomarkers of brain injury were increased in patients with impaired CVR in all intervention groups during the first 12 h but not at later time points.

## Discussion

In this post-hoc analysis of the COMACARE study cohort focusing on cerebrovascular reactivity (CVR), a majority of patients had impaired reactivity as assessed by the tissue oxygenation index (TOx), most commonly in patients with chronic hypertension. The TOx was higher, consistent with impaired CVR, in patients with poor neurological outcome over the whole time period studied (0–48 h) but not in the isolated 12–24 h intervals. Patients with poor neurological outcome had a decreased upper MAP bound and a narrower MAP range for maintained CVR but there was no difference in the lower MAP bound. The decreased upper MAP bound for maintained CVR in patients with poor outcome was observed in patients treated with a higher rather than lower oxygen, carbon dioxide and MAP targets when the subgroups were studied separately. Patients with impaired CVR had increased levels of the brain injury biomarker NfL but not NSE or S100b.

The importance of optimising cerebral oxygen delivery to mitigate hypoxic-ischaemic brain injury is supported by observational data that include strategies of augmented MAP to ameliorate ongoing cerebral ischemia in patients after cardiac arrest [6]. Previous smaller studies have demonstrated that impaired CVR is associated with hypoxic-ischaemic brain injury and poor neurological outcomes following cardiac arrest (CA) [4, 5, 15]. Regulatory interdependence between MAP and arterial blood gases provides synergistic effects on CBF and underpins the rationale of this post-hoc analysis. While the TOx in the isolated time periods studied was not statistically different between patients with good and poor neurological outcomes, the time-integrated TOx was higher consistent with impaired CVR in patients with poor neurology. Ameloot et al. [13] studied 51 comatose survivors of non-traumatic CA treated with target temperature management (TTM) at 33 °C for 24 h and reported impaired CVR in 35% of the patients using the same cut-off for the slope of the cStO2/MAP correlation (> 0) as used in this study. Pham et al. [12], using a similar TO_x_ definition for impaired CVR, reported an incidence rate of 15/23 (65%) in comatose survivors of CA of cardiac origin, which is very similar to the proportion found in the present study, which comprised almost five times as many subjects. Sekhon et al. [14] performed a feasibility study of TO_x_ in 20 cardiac arrest patients and reported impaired CVR in 15% of all individual measurements using a higher threshold at TO_x_ > 0.3. Both Ameloot et al. and Pham et al. demonstrated an association between CVR and clinical outcomes, either as the odds’ ratio for survival in a good neurological state in patients with maintained CVR (4.62 [1.06–20.1]) [5] or as the odds ratio for survival in patients with impaired CVR (0.15 [0.01–0.50]) [4]. The variability in CBF attributable to changes in MAP may be limited in critically ill patients [23] hence disrupting the coherence of vascular pressure reactivity to assess CVR. This could explain the lack of association between TOx in isolated time periods and neurological outcomes while the time-integrated TOx might represent a more robust measure of CVR after CA. These findings indicate that the duration of data capture warrants careful consideration in the design of any TOx guided interventional studies in CA patients as the most beneficial time for monitoring and therapy remain to be established. Both age and chronic hypertension demonstrated an independent association with impaired CVR in this study that is aligned with the higher frequency of disturbed CVR reported by Ameloot et al. [13] in patients with chronic hypertension. The importance of CVR is further highlighted by the increased NfL levels in patients with impaired CVR, indicating a more severe brain injury when CVR is attenuated.

Overall, cerebrovascular reactivity and the factors affecting it are regrettably still poorly known [24]. Previously, modification and optimization of CVR in brain injury and ICH by altering blood pressure and ventilation have been described in the literature [25, 26]. It has also been hypothesized that the brain endothelin systems could be used as therapeutic targets for improving CVR, as they are involved in both vasoconstriction and vasodilation of cerebral blood vessels, both key elements in CVR and also involved in pathologic processes leading to both ischemia and oedema in brain injuries [27]. The TO_x_ analyses in this study allow for a more nuanced assessment of CVR capacity beyond a dichotomising threshold value. This includes the lower and upper MAP bounds and the range of MAP that delineates maintained CVR as well as the MAP associated with the lowest optimal TO_x_. This study used a more conservative threshold of TOx at 0.3 to delineate the lower and upper MAP bounds [12, 15] compared to previous studies that used the transition from negative to positive TO_x_ [4, 5]. The upper MAP bound for maintained CVR was consistently lower in patients with poor neurological outcomes than in those with good outcomes in all time intervals, and this included all the 2^3^ factorial design groups with different targets for MAP, PaO_2_ and PaCO_2_. The upper MAP bound was high at 95–100 mmHg in the whole study cohort, and while not statistically different by outcome in all intervention subgroups, the numerical contrasts were consistent. Furthermore, the MAP range for maintained CVR was narrower in patients with poor outcomes during all time intervals and in all subgroups of MAP, P_a_O_2_ and P_a_CO_2_. This is aligned with animal studies of increased intracranial pressure [28] or induced subarachnoidal haemorrhage [29] demonstrating decreased upper MAP bound and a narrower MAP range for CVR. It is furthermore supported by clinical data in postoperative cardiac surgical patients demonstrating that a decreased upper MAP bound and narrower range was associated with delirium in ICU [30] and studies in patients with traumatic brain injury showing that supra-optimal MAP was associated with worse neurological outcome [20, 31]. It is possible that the decreased upper MAP bound observed in this study was the result of increased intracranial pressure and/or transient hypoperfusion although this was not independently investigated. Taken together, these findings demonstrate a limited tolerability to MAP fluctuations in patients with an ultimately poor neurological outcome and suggest that a more individualised approach to MAP management after OHCA is needed [32]. Rather than presenting only a sensitivity to low MAP, this study indicates the need to limit excessively high MAP in patients with impaired CVR. This is not unexpected since the normal autoregulatory capacity of CBF is more efficacious at buffering increases in blood pressure [7, 33] suggesting the particular harm of high MAP when CVR is impaired.

Current guidelines on post-cardiac arrest care recommend targeting a MAP higher than 65 mmHg and using adequate diuresis (more than 0.5 ml/kg) and lactate clearance as a guide of adequate tissue perfusion [34]. The lower MAP bound for maintained CVR was very close to the 70 mmHg reported in a recent review [35], albeit not statistically different between patients with good or poor outcomes. Importantly, the lower MAP bound was significantly higher [7] than classically suggested [36]. The most effective point of CVR, OptTOx, was numerically lower and the associated OptMAP numerically higher in patients with good compared to those with poor neurological outcome. However, this difference did not attain statistical significance (*p* = 0.08) in contrast to previous studies that demonstrated lower OptMAP in patients with good outcomes [4, 5]. Ameloot et al. demonstrated that the time below the OptMAP was negatively associated with survival, although the effect estimate was moderate with an odds ratio of 0.97 (0.96–0.99) per % of total study time. Differences in study design and analysis might explain these divergent results. This study captured data continuously over a longer period (48 h vs 24 h) [5] and with considerably greater detail compared to the daily median of 81 min for the first three days [4]. Reports of a gradual return of CBF over 72 h after an initial reduction in the first 12 h of post-cardiac arrest [3, 37–39], with the present study encompassing a larger proportion of the latter phase, might confound comparisons to a dichotomous separation of outcomes dependent on the first 24 h of cerebral haemodynamics.

To answer the key question whether, and to what degree, cardiac arrest results in a disturbance in CVR requires an adequate control group. A previous pilot study demonstrated a lack of statistical difference between TO_x_ during the first three days in post-cardiac arrest patients managed in the intensive care unit and healthy volunteers [12]. This type of comparison is challenged by the short period for measurements with rapid and marked changes, particularly increases, in MAP used to assess CVR [4, 40] in normal subjects. This is not likely to occur in sedated patients with extended monitoring and using protocolised titration to MAP targets. The CVR is more effective in buffering decreases in MAP that are more likely to occur in a population with acute cardiac dysfunction or as part of the post-cardiac arrest syndrome than increases in MAP [7]. Assessments of CVR are also dependent on the range and dynamics of MAP changes [33].

### Strengths and limitations

Strengths of the current study include a pre-specified protocol for the analyses and a relatively large and homogeneous patient sample. The extended period of monitoring up to 48 h is likely to have captured all phases of post-cardiac arrest cerebral haemodynamics. Important limitations of NIRS and TOx to monitor CVR must be acknowledged. The use of NIRS to derive TO_x_ as a measure of CVR is dependent on the correlation between cStO_2_ and CBF, which might be variable [41]. Furthermore, different types of methods and mathematical approaches used to describe the CVR status, are still subject to several assumptions [42]. The use of NIRS for evaluation of CVR has nevertheless been widely studied and compared with these other metrics and with outcome in a large range of patient populations [13, 25, 43, 44]. In this study, resource use in the patients with intact and impaired CVR were not able to retrieve data on length of hospital stay and discharge destination even though this was included in the published protocol paper.

Perfusion pressure determined by MAP predominantly affects flow in the major cerebral arteries along with neurogenic control while other factors inherent to CVR such as blood CO2 and O2 tension, perivascular pH and metabolic neurovascular coupling affect the cerebral microcirculation at a level closer to the monitoring of cStO2 [7]. Barriers to oxygen diffusion after cardiac arrest may lead to dissociation between CBF and cStO2 [45]. The TOx variable has not been clinically validated in cardiac arrest patients but shows promise in patients at risk for adverse neurological events [13, 46, 47] and correlates with other measures of CVR based on intracranial pressure monitoring or transcranial Doppler flowmetry [43, 46, 48, 49]. However the physiology of CVR is more complex than a simple linear correlation between MAP and CBF. Notably, the correlations between CVR and P_a_O_2_ and P_a_CO_2_ levels appear to have non-linear characteristics [7]. It is biologically plausible that considerable heterogeneity across the cerebral vasculature and the anatomical regions of the brain may exist that further confound assessments of CVR restricted to linear correlations or when a local cStO2 measurement is taken to represent global CBF. The numerical results for CVR may not be applicable to patients monitored with other NIRS equipment as proprietary algorithms and optode designs to avoid extracranial contamination of the signal [44] generate different results [40]. The retrospective, observational design of this study means that no causal inferences may be made, and the results should be viewed to demonstrate associations and generate hypotheses for future investigation with TOx a potential clinical heuristic [24]. Comparisons of CVR in the COMACARE study with a cohort of healthy volunteers, while intended in the published protocol [19], were abandoned because of significant and unreconcilable incongruities in monitoring time and resolution, NIRS equipment and the degree and origin of MAP variations. Resource use in the patients with intact and impaired CVR including length of mechanical ventilation and ICU stay were captured. We have compared and reported these in Table 1. We acknowledge that our protocol paper (1) also mentioned length of hospital stay in the hospital and discharge destination but unfortunately, we have not been able to retrieve this data reliably for all patients.

## Conclusion

Impaired CVR was common after cardiac arrest and occurred especially in patients with chronic hypertension. The tissue oxygenation index to assess CVR was only different between patients with good and poor neurological outcomes at six months when integrated over 48 h but not in shorter time periods. A decreased upper MAP bound and a narrower MAP range for maintained CVR was associated with poor neurological outcomes and impaired CVR was associated with higher levels of NfL, a novel marker of axonal brain injury.

## Supplementary Information

**Additional file 1.** The mixed linear analysis of OptTOx, OptMAP, upper and lower MAP bounds and MAP range for maintained CVR in the 23 factorial design groups.

## Data Availability

The dataset consisting of de-identified participants’ data is available from the corresponding author upon reasonable request.

## Abbreviations

OHCA: Out-of-hospital cardiac arrest
ROSC: Return of spontaneous circulation
MAP: Mean arterial pressure
NIRS: Near-infrared spectroscopy
cSto_2_: Cerebral tissue oxygenation
PaCO_2_: Arterial carbon dioxide tension
PaO_2_: Arterial oxygen tension
TOx: Tissue oxygenation index
CVR: Cerebrovascular reactivity
OptTOx: Optimal cerebrovascular reactivity
OptMAP: Mean arterial pressure at optimal cerebrovascular reactivity
NSE: Neuron-specific enolase
NfL: Neurofilament light
APACHE II: Acute illness and chronic health evaluation score II
