# Supplementary material for: Association of deranged cerebrovascular reactivity with brain injury following cardiac arrest: a post-hoc analysis of the COMACARE trial
Source: Crit Care. 2021 Sep 28;25:350. doi: 10.1186/s13054-021-03764-6 (PMC8477475; doi:10.1186/s13054-021-03764-6)
Supplement: Supplementary file 1 — Additional file 1. The mixed linear analysis of OptTOx, OptMAP, upper and lower MAP bounds and MAP range for maintained CVR in the 23 factorial design groups. [file 13054_2021_3764_MOESM1_ESM.docx]

**Supplementary Table 1.** OptTOx, OptMAP, upper and lower MAP bounds and MAP range for maintained CVR in the 2^3^ factorial design groups PaCO2 low-normal or high-normal, normoxia or moderate hyperoxia and low-normal or high-normal MAP during the three time periods of 0–12h, 12–24h and 24–48h, with good or poor neurologic outcome. Mixed linear analysis.

|  | Group | 0–12h | 12–24h | 24–48h | p-value |
| --- | --- | --- | --- | --- | --- |
| OptTOx |  |  |  |  |  |
| PaCO2 low | Poor outcome  Good outcome | -0.21 (-0.36– -0.03)  -0.22 (-0.36– -0.10) | -0.33 (-0.46– -0.16)  -0.24 (-0.43– -0.13) | -0.17 (-0.38– -0.05)  -0.28 (-0.39– -0.15) | 0.444 |
| PaCO2 high | Poor outcome  Good outcome | -0.24 (-0.33– -0.02)  -0.27 (-0.38– -0.17) | -0.12 (-0.20–0.00)  -0.30 (-0.52– -0.10) | -0.21 (-0.35– -0.10)  -0.20 (-0.35– -0.09) | 0.095 |
| PaO2 low | Poor outcome  Good outcome | -0.18 (-0.27– -0.04)  -0.26 (-0.39– -0.14) | -0.33 (-0.39– -0.12)  -0.27 (-0.44– -0.12) | -0.18 (-0.38– -0.07)  -0.27 (-0.41– -0.14) | 0.179 |
| PaO2 high | Poor outcome  Good outcome | -0.25 (-0.36–0.06)  -0.22 (-0.36– -0.13) | -0.14(-0.32–0.04)  -0.32 (-0.50– -0.09) | -0.26 (-0.36– -0.02)  -0.19 (-0.33– -0.09) | 0.342 |
| MAP low | Poor outcome  Good outcome | -0.24 (-0.39– 0.05)  -0.24 (-0.36– -0.13) | -0.14 (-0.34–0.02)  -0.26 (-0.52– -0.12) | -0.18 (-0.33– -0.05)  -0.20 (-0.37– -0.11) | 0.120 |
| MAP high | Poor outcome  Good outcome | -0.21 (-0.29– -0.08)  -0.29 (-0.43– -0.14) | -0.23 (-0.41– -0.07)  -0.29 (-0.37– -0.14) | -0.30 (-0.40– -0.05)  -0.24 (-0.38– -0.11) | 0.366 |
| OptMAP |  |  |  |  |  |
| PaCO2 low | Poor outcome  Good outcome | 78.8 (73.3–88.7)  80.9 (73.2–86.7) | 78.2 (71.0–82.7)  82.5 (73.0–87.0) | 80.0 (73.1–88.4)  83.2 (76.6–89.0) | 0.355 |
| PaCO2 high | Poor outcome  Good outcome | 80.8 (73.2–87.8)  80.9 (71.4–91.4) | 74.1 (71.5–80.6)  80.2 (70.1–91.7) | 81.9 (72.4–87.9)  82.1 (73.7–86.4) | 0.136 |
| PaO2 low | Poor outcome  Good outcome | 79.2 (72.8–88.7)  81.6 (73.2–89.3) | 76.4 (70.7–79.6)  81.7 (72.8–88.4) | 81.9 (72.6–88.7)  83.2 (75.0–87.9) | 0.333 |
| PaO2 high | Poor outcome  Good outcome | 79.7 (73.8–87.1)  79.0 (71.4–88.5) | 76.4 (71.3–81.9)  81.2 (71.5–86.8) | 78.4 (72.7–86.9)  82.6 (75.0–86.3) | 0.160 |
| MAP low | Poor outcome  Good outcome | 75.3 (71.6–79.7)  73.2 (69.4–79.3) | 72.8 (70.2–76.5)  73.3 (69.8–81.1) | 76.9 (69.3–86.3)  76.4 (72.4–82.7) | 0.636 |
| MAP high | Poor outcome  Good outcome | 88.0 (81.3–93.7)  86.7 (81.7–91.9) | 81.8 (76.8–88.4)  87.0 (82.2–92.1) | 81.9 (74.6–89.0)  86.4 (83.3–90.0) | **0.032** |
| Upper MAP bound |  |  |  |  |  |
| PaCO2 low | Poor outcome  Good outcome | 89.5 (80.2–100.7)  93.8 (85.1–104.9) | 92.2 (81.4–95.4)  93.4 (83.4–98.9) | 92.1 (84.7–97.8)  96.1 (90.5–101.3) | 0.059 |
| PaCO2 high | Poor outcome  Good outcome | 91.2 (82.2–95.5)  94.8 (86.8–101.3) | 83.8 (78.3–90.3)  91.1 (83.2–103.0) | 92.8 (82.3–101.4)  93.7 (88.5–100.5) | **0.012** |
| PaO2 low | Poor outcome  Good outcome | 92.4 (82.0–100.7)  94.8 (85.6–102.4) | 90.1 (82.1–92.7)  91.8 (82.4–99.8) | 94.9 (88.4–101.6)  94.3 (90.5–101.0) | 0.179 |
| PaO2 high | Poor outcome  Good outcome | 88.5 (80.3–95.5)  94.7 (85.1–104.5) | 88.4 (76.6–93.5)  94.5 (84.3–101.7) | 85.7 (82.3–96.6)  95.2 (88.7–100.7) | **0.002** |
| MAP low | Poor outcome  Good outcome | 84.7 (80.3–91.5)  85.9 (83.4–93.8) | 82.0 (79.2–89.7)  86.5 (81.0–92.5) | 91.6 (82.2–98.0)  90.6 (87.1–95.2) | 0.089 |
| MAP high | Poor outcome  Good outcome | 97.2 (90.5–101.5)  102.0 (95.9–108.8) | 92.8 (88.9–95.9)  99.8 (95.7–106.8) | 93.8 (86.6–100.9)  100.0 (93.4–105.1) | **0.001** |
| Lower MAP bound |  |  |  |  |  |
| PaCO2 low | Poor outcome  Good outcome | 70.5 (64.0–77.2)  66.9 (63.1–75.6) | 67.5 (61.8–71.8)  69.7 (62.4–74.6) | 70.3 (63.7–78.3)  68.6 (64.5–73.2) | 0.703 |
| PaCO2 high | Poor outcome  Good outcome | 68.0 (64.9–73.9)  72.3 (64.4–76.9) | 64.9 (63.0–74.3)  64.9 (60.1–75.0) | 65.7 (62.1–71.8)  68.9 (61.8–73.5) | 0.399 |
| PaO2 low | Poor outcome  Good outcome | 66.7 (63.8–80.1)  68.0 (64.0–76.4) | 63.7 (61.4–73.1)  64.5 (60.5–76.2) | 69.1 (61.3–75.2)  68.4 (64.1–72.3) | 0.658 |
| PaO2 high | Poor outcome  Good outcome | 70.0 (65.7–73.5)  70.9 (62.8–76.9) | 67.5 (63.2–70.7)  70.8 (62.8–74.1) | 66.9 (63.5–74.1)  69.4 (61.8–73.6) | 0.660 |
| MAP low | Poor outcome  Good outcome | 65.7 (61.6–69.6)  64.0 (60.9–66.0) | 63.5 (60.6–66.1)  62.2 (59.2–64.4) | 64.8 (61.1–70.3)  64.1 (60.6–67.7) | 0.106 |
| MAP high | Poor outcome  Good outcome | 75.7(70.8–80.1)  75.9 (73.3–79.5) | 73.1 (67.7–80.3)  74.6 (71.9–79.2) | 71.4 (65.7–76.0)  73.5 (70.2–75.4) | 0.165 |
| MAP range | Poor outcome  Good outcome | 18.9 (15.1–30.2)  28.0 (22.9–35.8) | 23.6 (17.3–26.0)  25.0 (20.0–36.1**)** | 22.5 (17.0–29.1)  33.0 (25.1–38.6) | **<0.001** |
| PaCO2 low | Poor outcome  Good outcome | 19.6 (14.3–28.3)  30.9 (22.5–40.7) | 24.2 (21.0–28.6)  27.9 (20.7–38.1) | 22.3 (11.9–28.2)  32.3 (24.6–38.4) | **0.001** |
| PaCO2 high | Poor outcome  Good outcome | 18.9 (15.4–30.7)  27.9 (24.4–34.9) | 23.6 (15.6–25.3)  24.2 (20.5–34.1) | 23.9 (19.1–39.6)  33.6 (25.9–38.7) | **0.008** |
| PaO2 low | Poor outcome  Good outcome | 19.6 (14.4–31.8)  28.0 (22.9–40.1) | 25.3 (21.7–25.4)  27.6 (20.5–37.7) | 27.7 (15.9–47.9)  31.5 (25.1–38.6) | **0.038** |
| PaO2 high | Poor outcome  Good outcome | 18.9 (15.4–27.1)  28.8 (21.6–35.8) | 21.5 (14.6–29.7)  27.3 (20.7–33.6) | 22.0 (17.8–24.3)  33.9 (23.6–38.6) | **<0.001** |
| MAP low | Poor outcome  Good outcome | 23.8 (14.4–31.7)  28.0 (22.9–34.9) | 25.3 (15.3–26.5)  27.8 (21.2–37.1) | 22.9 (15.3–35.5)  31.6 (27.9–39.1) | **0.002** |
| MAP high | Poor outcome  Good outcome | 17.7 (15.4–23.2)  30.0 (22.5–40.7) | 22.4 (17.6–26.7)  24.1 (20.0–34.7) | 22.5 (20.0–28.4)  33.9 (23.4–38.3) | **0.004** |

Values are expressed as medians (interquartile ranges).

**Supplementary Table 2.** Time-integrated mean arterial pressure (MAP) area (mm Hg **.** min) between the lower and upper MAP bounds for maintained cerebrovascular reactivity (CVR) and total MAP area below the lower and above the upper MAP bound for maintained CVR stratified by the factorial design groups. P-values represent mixed linear analysis of poor six-month neurologic outcomes (CPC1–2 or 3–5).

|  | Group | 0–12h | 12–24h | 24–48h | p-value |
| --- | --- | --- | --- | --- | --- |
| MAP area between the lower and upper MAP bounds, mmHg **.** min |  |  |  |  |  |
| PaCO2 low | Poor outcome  Good outcome | 4527 (2897–6793)  6984 (5523–8292) | 6903 (5699–10494)  8389 (5665–11481) | 5456 (3851–12139)  9830 (4828–17288) |  |
| PaCO2 high | Poor outcome  Good outcome | 5550.4 (3442.2–7813.9)  6146.3 (4559.4–9864.7) | 5286.0 (2970.0–8026.1)  8500.5 (4750.9–11636.6) | 12245 (9466–18861)  16878 (11120–21512) | 0.629 |
| PaO2 low | Poor outcome  Good outcome | 5786 (4833–7958)  6757 (5014–8467) | 6449 (5769–9421)  8507 (6007–11461) | 12000 (6207–26142)  17867 (10093–22684) | 0.901 |
| PaO2 high | Poor outcome  Good outcome | 4364 (2097–6695)  7160 (4197–9315) | 5286 (2735–9276)  8087 (4492–11870) | 12315 (6185–15785)  16766 (10730–23154) | 0.110 |
| MAP low | Poor outcome  Good outcome | 5542 (2724–8388)  6948 (5142–8302) | 6903 (3495–10520)  8118 (4758–11878) | 12000 (6207–16705)  17175 (8794–22972) | 0.527 |
| MAP high | Poor outcome  Good outcome | 5345 (3437–6404)  6900 (4274–10122) | 5370 (3244–7608)  8998 (6141–11130) | 13103 (8648–17543)  17283 (11996–22289) | 0.425 |
| MAP area below the lower MAP bound, mmHg **.** min |  |  |  |  |  |
| PaCO2 low | Poor outcome  Good outcome | 76 (28–464)  127 (41–397) | 120 (60–523)  46 (24–213) | 507 (102–3210)  235 (53–439) | **0.024** |
| PaCO2 high | Poor outcome  Good outcome | 320 (78–764)  94 (17–423) | 179 (41–494)  151 (75–523) | 147 (75–921)  248 (57–758) | 0.688 |
| PaO2 low | Poor outcome  Good outcome | 101 (40–322)  85 (27–185) | 94 (20–355)  111 (53–306) | 136 (69–1351)  235 (70–761) | 0.561 |
| PaO2 high | Poor outcome  Good outcome | 334 (76–775)  237 (41–818) | 189 (69–426)  73 (25–347) | 306 (89–1186)  224 (25–463) | 0.083 |
| MAP low | Poor outcome  Good outcome | 144 (46–775)  35 (22–197) | 105 (16–535)  73 (24–270) | 146 (84–958)  159 (20–774) | 0.850 |
| MAP high | Poor outcome  Good outcome | 232 (54–446)  219 (94–657) | 157 (77–218)  126 (30–333) | 246 (64–3300)  322 (80–491) | **0.021** |
| MAP area above the upper MAP bound, mmHg **.** min |  |  |  |  |  |
| PaCO2 low | Poor outcome  Good outcome | 315 (102–1635)  395 (96–1162) | 137 (53–498)  108 (26–232) | 383 (79–1142)  287 (83–766) | 0.683 |
| PaCO2 high | Poor outcome  Good outcome | 313 (102–1363)  249 (80–565) | 579 (98–4899)  219 (38–2117) | 271 (148–692)  161 (43–1847) | 0.494 |
| PaO2 low | Poor outcome  Good outcome | 504 (75–3049)  344 (119–1162) | 257 (59–4176)  162 (28–324) | 202 (138–542)  203 (87–989) | 0.089 |
| PaO2 high | Poor outcome  Good outcome | 302 (113–1057)  180 (54–616) | 252.5 (75.0–1106.8)  76.1 (14.0–4024.3) | 393 (167–1256)  274 (54–1045) | 0.951 |
| MAP low | Poor outcome  Good outcome | 238 (81–1389)  160 (78–495) | 256 (61–4853)  56 (21–525) | 403 (202–1871)  363 (143–1847) | 0.388 |
| MAP high | Poor outcome  Good outcome | 518 (119–1698)  395 (98–1004) | 249 (69–7485)  192 (67–333) | 173 (102–504)  178 (48–892) | 0.733 |

Values are expressed as medians (interquartile ranges).

**Supplementary Table 3.** NSE, S100B and NfL levels a 48h after OHCA stratified by the factorial design groups.

|  | Autoreg intact | Autoreg impaired | p-value |
| --- | --- | --- | --- |
| NSE 48h |  |  |  |
| PaCO2 low | 18.2 (13.5–23.3) | 24.9 (14.9–34.9) | 0.148 |
| PaCO2 high | 23.5 (13.4–35.2) | 22.6 (14.7–35.7) | 0.871 |
| PaO2 low | 18.8 (13.4–24.6) | 25.2 (15.5–32.6) | 0.105 |
| PaO2 high | 18.8 (14.3–33.6) | 21.6 (13.7–37.3) | 0.489 |
| MAP low | 18.8 (13.9–32.3) | 22.3 (15.1–35.5) | 0.553 |
| MAP high | 21.6 (13.5–31.0) | 25.0 (14.2–35.2) | 0.097 |
| S100B 48h |  |  |  |
| PaCO2 low | 0.06 (0.05–0.08) | 0.10 (0.07–0.18) | **0.001** |
| PaCO2 high | 0.08 (0.06–0.12) | 0.08 (0.06–0.19) | 0.239 |
| PaO2 low | 0.06 (0.04–0.09) | 0.09 (0.07–0.18) | **0.013** |
| PaO2 high | 0.08 (0.06–0.11) | 0.09 (0.06–0.19) | 0.585 |
| MAP low | 0.07 (0.05–0.11) | 0.09 (0.07–0.19) | 0.103 |
| MAP high | 0.06 (0.05–0.09) | 0.09 (0.06–0.22) | 0.237 |
| NfL 48h |  |  |  |
| PaCO2 low | 17.8 (13.0–199.3) | 31.6 (19.9–208.3) | 0.125 |
| PaCO2 high | 20.8 (16.9–680.9) | 57.2 (12.2–1712.9) | 0.308 |
| PaO2 low | 21.9 (13.9–520.5) | 29.7 (14.8–264.0) | 0.606 |
| PaO2 high | 19.1 (11.7–321.4) | 54.9 (16.6–2642.3) | 0.120 |
| MAP low | 24.2 (14.5–657.9) | 43.8 (22.8–2263.7) | 0.228 |
| MAP high | 19.1 (9.7–32.8) | 30.9 (11.0–447.2) | 0.251 |

**Supplementary Table 4.** NSE 48h, S100B and NfL after OHCA analysed in the high or low carbon dioxide, oxygen and MAP groups indexed by mean TOx during 0–12h, 12–24h and 24–48h.

|  | 0–12h | 0–12h |  | 12–24h | 12–24h |  | 24–48h | 24–48h |  |
| --- | --- | --- | --- | --- | --- | --- | --- | --- | --- |
|  |  |  |  |  |  |  |  |  |  |
|  | Mean TOx<0 | Mean TOx>0 | p-value | Mean TOx<0 | Mean TOx>0 | p-value | Mean TOx<0 | Mean TOx>0 | p-value |
| NSE 48h |  |  |  |  |  |  |  |  |  |
| PaCO2 low | 18.2 (13.8–26.4) | 25.8 (15.5–59.3) | 0.067 | 17.5 (13.5–26.3) | 25.8 (16.2–29.5) | **0.037** | 19.1 (15.0–25.7) | 25.9 (15.5–54.5) | 0.142 |
| PaCO2 high | 20.6 (13.7–30.0) | 30.1 (15.1–76.8) | 0.058 | 22.3 (13.4–36.9) | 22.6 (14.8–33.3) | 0.574 | 25.0 89 (17.3–36.9) | 20.0 (13.8–31.8) | 0.282 |
| PaO2 low | 18.9 (13.7–24.8) | 27.5 (20.9–63.6 | **0.009** | 21.9 (13.9–34.1) | 24.3 (15.0–27.7) | 0.954 | 22.3 (15.0–30.5) | 25.5 (15.5–30.3) | 0.437 |
| PaO2 high | 18.3 (13.7–30.2) | 23.9 (14.4–86.3) | 0.174 | 17.4 (13.5–33.0) | 27.4 (14.8–34.9) | 0.055 | 23.4 (14.8–33.0) | 17.3 (13.6–34.9) | 0.905 |
| MAP low | 18.8 (14.6–26.5) | 25.4 (15.0–81.1) | 0.301 | 18.7 (13.4–30.6) | 24.0 (15.5–32.6) | 0.164 | 19.4 (16.4–30.5) | 21.0 (14.9–35.1) | 0.883 |
| MAP high | 17.4 (13.5–27.2) | 28.3 (19.9–67.6) | **0.006** | 21.6 (13.5–33.8) | 25.0 (14.5–30.7) | 0.544 | 24.4 (14.6–33.6) | 21.6 (13.8–30.3) | 0.630 |
| S100B 48h |  |  |  |  |  |  |  |  |  |
| PaCO2 low | 0.08 (0.06–0.10) | 0.11 (0.06–0.26) | **0.009** | 0.07 (0.06–0.11) | 0.09 (0.08–0.12) | **0.044** | 0.08 (0.06–0.10) | 0.10 (0.07–0.20) | **0.005** |
| PaCO2 high | 0.08 (0.06–0.11) | 0.09 (0.06–0.30) | 0.245 | 0.09 (0.06–0.15) | 0.08 (0.06–0.12) | 0.919 | 0.09 (0.06–0.15) | 0.08 (0.06–0.17) | 0.279 |
| PaO2 low | 0.08 (0.06–0.10) | 0.11 (0.06–0.26) | 0.068 | 0.07 (0.05–0.18) | 0.09 (0.06–0.10) | 0.881 | 0.07 (0.06–0.10) | 0.09 (0.07–0.18) | 0.070 |
| PaO2 high | 0.08 (0.06–0.11) | 0.11 (0.07–0.30) | 0.060 | 0.08 (0.06–0.11) | 0.09 (0.07–0.19) | 0.213 | 0.09 (0.07–0.12) | 0.09 (0.06–0.19) | 0.591 |
| MAP low | 0.09 (0.06–0.10) | 0.11 (0.07–0.24) | **0.044** | 0.09 (0.06–0.12) | 0.09 (0.06–0.11) | 0.685 | 0.09 (0.06–0.11) | 0.09 (0.07–0.19) | 0.337 |
| MAP high | 0.08 (0.06–0.10) | 0.11 (0.06–0.34) | 0.125 | 0.08 (0.06–0.14) | 0.09 (0.06–0.12) | 0.461 | 0.08 (0.06–0.12) | 0.08 (0.06–0.17) | 0.888 |
| NfL 48h |  |  |  |  |  |  |  |  |  |
| PaCO2 low | 22.6 (13.0–43.2) | 48.0 (22.0–2964.2) | **0.007** | 28.4 (13.8–95.7) | 29.6 (16.5–48.6) | 0.552 | 19.7 (13.6–38.5) | 43.4 (23.5–1928.0) | **0.025** |
| PaCO2 high | 21.8 (9.7–63.3) | 447.2 (15.2–4473.1) | **0.008** | 23.1 (15.2–1033.4) | 51.4 (11.2–468.1) | 0.359 | 22.8 (11.0–1033.4) | 47.4 (13.2–924.8) | 0.762 |
| PaO2 low | 22.8 (14.0–33.9) | 45.3 (16.4–2455.1) | **0.042** | 27.8 (13.9–686.7) | 25.1 (12.9–57.0) | 0.578 | 22.8 (12.4–155.9) | 36.6 (21.9–410.9) | 0.154 |
| PaO2 high | 19.1 (9.7–47.6) | 245.8 (22.8–4712.8) | **0.001** | 23.1 (14.7–133.3) | 54.9 (13.6–2080.1) | 0.246 | 19.7 (12.6–204.2) | 47.6 (15.3–1967.1) | 0.283 |
| MAP low | 27.2 (14.9–60.5) | 239.4 (27.6–3711.7) | **0.008** | 29.1 (16.3–419.0) | 53.3 (15.1–924.8) | 0.388 | 28.4 (14.9–419.0) | 43.6 (26.4–1967.1) | 0.204 |
| MAP high | 16.8 (9.2–31.7) | 45.3 (16.1–2455.1) | **0.006** | 20.8 (10.0–463.4) | 30.7 (12.8–54.9) | 0.992 | 18.9 (9.8–36.3) | 38.1 (11.8–396.8) | 0.216 |
